# Harnessing glycerol for secondary metabolite biosynthesis in microorganisms

**DOI:** 10.1007/s11274-025-04537-x

**Published:** 2025-09-15

**Authors:** Kouhei Kamasaka, Luiz Marcello, Lucília Domingues, Tomohisa Hasunuma

**Affiliations:** 1 Engineering Biology Research Center, Kobe University, 1-1 Rokkodai, Nada, Kobe, 657-8501 Japan; 2 Graduate School of Science, Technology and Innovation, Kobe University, 1- 1 Rokkodai, Nada, Kobe, 657-8501 Japan; 3 Centre of Biological Engineering, University of Minho, Braga, 4710-057 Portugal; 4 LABBELS – Associate Laboratory, Braga/Guimarães, Portugal

**Keywords:** Glycerol, Secondary metabolite, Metabolic engineering, Crude glycerol valorization, Biodiesel byproduct

## Abstract

The increase in glycerol availability as a byproduct of biodiesel production has attracted attention in its utilization as a carbon source for microbial fermentation. Compared with traditional carbon sources such as glucose, glycerol enhances the production of various secondary metabolites such as aromatic compounds, polyols, and lipids in microorganisms such as *Komagataella phaffii* and *Yarrowia lipolytica*. For instance, in *K. phaffii*, glycerol increased the titers of *p*-coumarate, naringenin, and resveratrol compared with glucose-based media, particularly when combined with specific amino acid supplementation. This review presents recent advancements in the production of secondary metabolites from glycerol. The metabolic pathways involved in glycerol utilization and secondary metabolite biosynthesis have been examined with insights into the regulation of these processes. The potential applications of glycerol-derived secondary metabolites in the pharmaceutical, food, and biofuel industries have been discussed. Finally, the challenges such as strain robustness, variability in crude glycerol quality, and downstream processing have been addressed with focus on recent innovations in metabolic engineering, redox tuning, and bioprocess control. Overall, this review highlights the potential of glycerol in the context of these advancements as a sustainable and cost-effective feedstock for the next-generation microbial biomanufacturing of high-value secondary metabolites.

**Graphical abstract:**

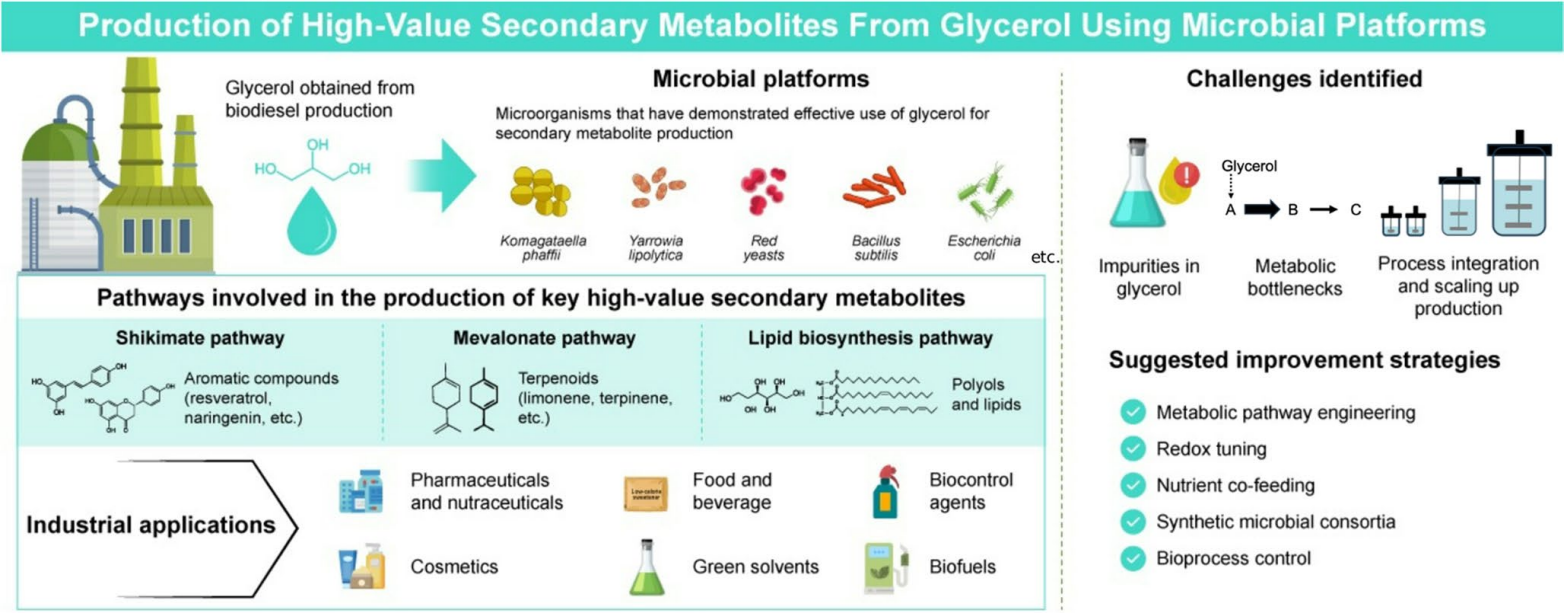

## Introduction

Secondary metabolites are referred to as specialized metabolites and are a diverse group of compounds synthesized by microorganisms, plants, and fungi (Reshi et al. 2023). Primary metabolites are essential for growth and reproduction, whereas secondary metabolites are not required for survival but to obtain ecological advantages such as defense, signaling, and competition (Ozyigit et al. 2023). Furthermore, secondary metabolites possess significant industrial value and are the basis for a wide array of pharmaceuticals (e.g., antibiotics and anticancer agents), nutraceuticals, food additives, cosmetics, pigments, and agricultural biocontrol agents.

The growing global demand for these high-value products has led to increased interest in microbial production systems owing to their scalability, specificity, and sustainability. Although plants are the traditional sources of secondary metabolites, the use of plants to produce secondary metabolites involves challenges such as long cultivation cycles, low yields, and susceptibility to environmental variability. In contrast, microbial fermentation enables continuous and controlled production in bioreactors using renewable substrates (Rusu et al. 2023; Kumokita et al. 2023).

Glycerol has garnered considerable attention as a renewable carbon source. Furthermore, glycerol is a by-product of biodiesel production and is generated at an estimated 10% of the total biodiesel volume (da Silva et al. 2009). The rapid expansion of the biodiesel industry worldwide has resulted in abundant glycerol availability, which has created both an opportunity and a challenge—although glycerol is a cost-effective and sustainable substrate for microbial fermentation, its surplus raises concerns of its disposal and valorization (Monteiro et al. 2018; Liu et al. 2022; Tomatis et al. 2024).

Chemically, glycerol is a trihydroxy alcohol (C₃H₈O₃) with a higher degree of reduction than that of glucose, making it a favorable substrate for the biosynthesis of reduced compounds such as lipids and polyols (Clomburg and Gonzalez 2013). Furthermore, it is metabolized by a broad range of microorganisms including bacteria, yeasts, and fungi (Santamaria et al. 2022). In several cases, glycerol has been reported to outperform glucose in supporting the production of valuable secondary metabolites—for instance, citric acid in *Yarrowia lipolytica* (Li and Townsend 2006), lovastatin in *Aspergillus terreus* (Subhan et al. 2016), and clavulanic acid in *Streptomyces clavuligerus* (Saudagar and Singhal 2007). However, efficient glycerol metabolism is species- and strain-dependent and is often limited by regulatory mechanisms, transport efficiency, and redox balance.

Over the past decade, microbial biotechnologists have increasingly explored the use of glycerol as a substrate, and it outperformed glucose in promoting the biosynthesis of specific compounds under nitrogen-limited conditions that trigger secondary metabolism (Rakicka et al. 2015; Sara et al. 2016; Vastaroucha et al. 2024). Despite these successes, several challenges remain such as the need to fully elucidate the partially elucidated glycerol metabolism-related regulatory pathways in several industrial hosts. Furthermore, glycerol is traditionally categorized as a nonfermentable carbon source, and the growth of the yeast *Saccharomyces cerevisiae* on glycerol is often slow and inefficient without significant genetic modifications (Swinnen et al. 2016). Additionally, the use of crude glycerol, which contains methanol, salts, and other impurities, requires careful pretreatment or strain adaptation (Samul et al. 2014).

The aim of this review is to provide a comprehensive overview of the recent progress in utilizing glycerol for microbial biosynthesis of secondary metabolites. The metabolic pathways involved in glycerol assimilation are examined, with key microbial platforms that have been engineered or selected for efficient production highlighted. Bioprocess strategies that maximize yields are analyzed. Additionally, the industrial relevance of the resulting compounds is discussed, and the knowledge gaps and future research directions for advancing this sustainable bioproduction strategy are identified.

## Glycerol as a substrate

Glycerol is also known as glycerin. It is a trihydroxy alcohol that has become an increasingly important feedstock in industrial biotechnology because of its abundance, low cost, and favorable chemical properties (da Silva et al. 2009; Tan et al. 2013; Poblete-Castro et al. 2020). It is a major byproduct of several industrial processes including biodiesel production, oleochemical operations such as saponification and fat hydrolysis, and bioethanol production. The exponential growth of the biodiesel industry has resulted in a corresponding increase in glycerol production with approximately 1 ton of glycerol generated for every 10 tons of biodiesel (da Silva et al. 2009). According to the OECD-FAO outlook (2022–2031), global biodiesel production is projected to reach 55 billion liters by 2031, resulting in an estimated 5.5 billion liters of crude glycerol (Organization for Economic Co-operation and Development and Food and Agriculture Organization 2022). This surplus often occurs in the form of low-purity crude glycerol and has created a need for innovative valorization strategies to convert this waste stream into value-added products (Sato 2021). Biochemically, glycerol exhibits several characteristics that make it a promising carbon source for microbial fermentation. Its higher degree of reduction compared with that of glucose enables higher NADH and NADPH generation during metabolism, which may be advantageous for the biosynthesis of reduced secondary metabolites such as lipids, polyols, and certain aromatic compounds (Xiberras et al. 2019). Furthermore, glycerol metabolism typically yields lower biomass than that of glucose because of the potential favoring of carbon flux allocation toward product formation rather than cell growth, which is an important consideration for secondary metabolite production that often occurs during the stationary phase (Sudarsan et al. 2025).

Microorganisms assimilate glycerol through one of two primary pathways: the dihydroxyacetone (DHA) pathway or glycerol 3-phosphate (G3P) pathway. In many yeasts and bacteria, glycerol is first phosphorylated to form G3P, which is oxidized to a glycolysis intermediate known as DHA phosphate (DHAP). Alternatively, glycerol is oxidized to DHA by glycerol dehydrogenase, followed by phosphorylation by DHA kinase. These pathways are integrated into the central carbon metabolism and influence the redox state of the cell, which impacts overall metabolic fluxes (da Silva et al. 2009; Xiberras et al. 2019).

Although glycerol is efficiently metabolized by some microbes, its use is limited in others. For example, *S. cerevisiae* has been historically considered inefficient in glycerol metabolism owing to its slow growth rate and low uptake capacity. However, Xiberras et al. (2019) have reported that these limitations may be attributed to the incomplete understanding of key metabolic nodes such as mitochondrial transport systems, glyoxylate shunt activity, and subcellular enzyme localization. This knowledge gap has hindered efforts to optimize *S. cerevisiae* for glycerol-based fermentation. Thus, systems-level studies using transcriptomics, proteomics, and fluxomics are needed to bridge this knowledge gap.

In contrast, non-conventional yeasts such as *Komagataella phaffii* (*Pichia pastoris*) and *Yarrowia lipolytica* exhibit robust glycerol metabolism and have been successfully used for the heterologous production of various secondary metabolites. Glycerol supports high cell densities in *K. phaffii* and allows the tight regulation of gene expression via the glycerol-induced AOX1 promoter system (Wang et al. 2017; Kumokita et al. 2022). *Y. lipolytica* efficiently channels glycerol for lipid biosynthesis and polyol production, particularly under nitrogen-limited conditions (Egermeier et al. 2017; Papanikolaou et al. 2017a; Rywińska et al. 2024).

Furthermore, several environmental parameters such as pH, temperature, and oxygen availability affect the utilization of glycerol in fermentation systems. Generally, aerobic conditions promote glycerol assimilation via oxidative pathways, enhance flux through central carbon metabolism, and support high biomass and metabolite yields. In contrast, oxygen-limited conditions may divert metabolism toward reductive branches, resulting in the accumulation of reduced products or fermentation intermediates (da Silva et al. 2009; Xiberras et al. 2019). Additionally, the choice of nitrogen and phosphorus sources, their concentrations, and timing of supplementation significantly influence metabolic flux distribution, biomass accumulation, and secondary metabolite profile and yield. For example, nitrogen limitation is well known to trigger the onset of secondary metabolism in several microbial systems such as polyol and lipid production in *Y. lipolytica* and antifungal metabolite production in *B. subtilis* (Trivunović et al. 2022).

Overall, glycerol is a promising and sustainable feedstock for secondary metabolite production, especially in the context of circular bioeconomic strategies for the valorization of industrial by-products. However, a detailed understanding of microbial glycerol metabolism, targeted strain engineering, and optimized bioprocess conditions tailored to specific product pathways is needed to harness its full potential in these areas.

### Microbial platforms for secondary metabolite production

The choice of microbial host critically influences the efficiency, yield, and diversity of secondary metabolites produced from glycerol. Key selection criteria include glycerol assimilation capabilities, tolerance to product toxicity, availability of genetic tools, and established fermentation protocols. This section reviews major microbial platforms—yeasts, bacteria, and filamentous fungi—that effectively convert glycerol into secondary metabolites. To facilitate comparison, detailed information on host strains, target metabolites and production levels are summarized in Table 1. Microbial species differ in their glycerol catabolic pathways, impacting redox balance, precursor availability, and metabolic flux distribution. Glycerol is metabolized mainly via two pathways: (1) the G3P pathway (glycerol kinase and G3P dehydrogenase) or (2) the DHA pathway (glycerol dehydrogenase and DHA kinase). The primary glycerol assimilation route for each organism discussed is indicated.

**Table 1:** Secondary metabolite production from glycerol in microorganisms

Microorganism	Secondary metabolite(s)	Metabolic pathway	Glycerol assimilation pathway	Production	Reference
*K. phaffii*	Resveratrol, Naringenin, *p*-Coumarate	Engineered	G3P	~ 0.2–0.3 g/L	Kumokita et al. 2022, 2023
*Y. lipolytica*	Limonene	Engineered	G3P/DHA	23.6 mg/L	Cao et al. 2016
*Y. lipolytica*	Erythritol, Mannitol	Native	G3P/DHA	> 500 mg/g DCW (lipid), ~ 60 g/L (erythritol)	Papanikolaou et al. 2017a; Rywińska et al. 2013
*R. toruloides*	Carotenoids, Lipids	Native	G3P	≤ 2.8 mg/g DCW	Peik et al. 2013
*B. subtilis*	Antifungal metabolites	Native	G3P	Not reported	Trivunović et al. 2022
*S. clavuligerus*	Clavulanic acid, Cephamycin C	Native	G3P	~ 1.3 g/L	Saudagar and Singhal 2007
*A. gossypii*	Orotic acid	Engineered	G3P	> 4 g/L	Silva et al. 2022
*A. terreus*	Lovastatin	Native	G3P	~ 39.6 mg/L	Subhan et al. 2016

### Komagataella phaffii

*Komagataella phaffii* is a non-conventional yeast extensively employed for recombinant protein production and metabolic engineering. It exhibits robust glycerol metabolism primarily via the G3P pathway, enabling high cell densities and effective induction under glycerol-limiting conditions through the tightly regulated AOX1 promoter (Celik and Calık 2012; Wang et al. 2017). Using ^13^C-metabolic flux analysis, Tomàs-Gamisans et al. (2019) showed efficient glycerol channeling through glycolysis and the TCA cycle with minimal glyoxylate shunt activity, leading to enhanced NADPH regeneration via NADP⁺-dependent isocitrate dehydrogenase. Although the pentose phosphate pathway exhibits minimal activity under glycerol conditions, the redirection of carbon flux through glycolysis and the TCA cycle contributes to cofactor regeneration and anabolic metabolism. This is supported by auxiliary NADPH-generating reactions, such as those involving NADP⁺-dependent isocitrate dehydrogenase, as described by Tomàs-Gamisans et al. (2019). This enhanced NADPH availability supports biosynthetic pathways under glycerol-fed conditions.

*K. phaffii* has been engineered to produce heterologous plant-derived secondary metabolites including resveratrol, naringenin, and *p*-coumarate. Introduction of feedback-resistant ARO4^K229L^ and ARO7^G141S^ alleles boosted precursor (PEP and E4P) availability, and supplementation with aspartate or tryptophan further increased titers, yielding 92 mg/L *p*-coumarate, 297 mg/L naringenin, and 452 mg/L resveratrol in glycerol-fed cultures (Kumokita et al. 2022, 2023). These examples illustrate the metabolic versatility of *K. phaffii* for engineering secondary metabolite production from glycerol.

### Yarrowia lipolytica

*Yarrowia lipolytica* is an oleaginous yeast utilizing both G3P and DHA pathways for glycerol metabolism, with the G3P pathway predominating under aerobic conditions. It efficiently converts glycerol into lipids, polyols, and organic acids, especially under nitrogen-limited conditions. Its metabolic flexibility and GRAS status make it highly suitable for industrial biotechnology (Rywińska et al. 2013).

Under specific conditions, *Y. lipolytica* produces lipids exceeding 50% dry cell weight (DCW), erythritol (60–80 g/L), mannitol (~ 40 g/L), and citric acid (> 100 g/L) from pure and crude glycerol (Beopoulos et al. 2008; Sara et al. 2016; Papanikolaou et al. 2017b; Ziuzia et al. 2023). Although lipids and citric acid are considered primary metabolites, erythritol and mannitol function as hybrid metabolites with dual primary metabolic origins and secondary roles, such as osmotic stress tolerance and redox balance maintenance. Additionally, genetic engineering efforts have enabled *Y. lipolytica* to synthesize terpenoids like limonene. Cao et al. (2016) engineered a strain by overexpressing mevalonate (MVA) pathway genes (*HMG1*, *IDI1*, *ERG8*) and a limonene synthase from *Mentha* sp., resulting in a titer of 23.6 mg/L limonene—a 226-fold increase over baseline strain—in a two-phase cultivation system. This setup used a dodecane overlay to capture volatile limonene and reduce feedback inhibition. Cultivations were carried out in shake flasks with 20 mL working volume in 100 mL baffled flasks under aerobic conditions. These characteristics position *Y. lipolytica* as a versatile platform for sustainable metabolite production from glycerol.

### Saccharomyces cerevisiae

Although a cornerstone of industrial biotechnology, *S. cerevisiae* historically shows limited glycerol metabolism through its native G3P pathway (Nevoigt 2008). To our knowledge, reports of producing plant-derived secondary metabolites using *S. cerevisiae* with glycerol are very limited (Nan et al. 2020). Recent metabolic engineering, however, significantly enhanced glycerol utilization by introducing a heterologous DHA pathway alongside the native route, enabling efficient glycerol metabolism and ethanol production as proof of concept (Perpelea et al. 2025).

Under glycerol-fed conditions, the high degree of reduction of glycerol enhances the regeneration of redox cofactors, particularly NADPH, which is essential for these biosynthetic pathways. Redox-balancing strategies—including the use of NAD⁺-dependent glycerol dehydrogenases and NADH-regeneration systems—have also been employed to minimize the accumulation of inhibitory intermediates (Kim et al. 2017; Aßkamp et al. 2019). Furthermore, transporter engineering, such as the heterologous expression of aquaglyceroporins from glycerol-efficient yeasts (*K. phaffii*, *Y. lipolytica*, *Cyberlindnera jadinii*), has improved glycerol uptake, resulting in 30–40% higher specific growth rates and biomass yields (Klein et al. 2016). These advancements might be transforming *S. cerevisiae* into a versatile platform for glycerol-based bioproduction.

### Red yeasts

Red yeasts, notably *Rhodosporidium toruloides*, exhibit efficient glycerol metabolism via the G3P pathway, leading to high production of secondary metabolites such as carotenoids (torulene, torularhodin, β-carotene). Under high-carbon and low-nitrogen conditions, *R. toruloides* accumulates carotenoids and lipids (single-cell oils) exceeding 50% DCW (Papanikolaou et al. 2017a). Similarly, *Rhodotorula glutinis* and *Sporobolomyces shibatanus* grow robustly on crude glycerol, yielding up to 2.8 mg/g DCW carotenoids without detoxification (Petrik et al. 2013). These yeasts represent naturally productive platforms, advantageous due to their simplicity, robustness, and regulatory compatibility.

### Bacillus subtilis

*Bacillus subtilis* metabolizes glycerol via the G3P pathway, and is notable for synthesizing bioactive secondary metabolites, including antifungal lipopeptides such as surfactin, fengycin, iturin. Trivunović et al. (2022) optimized glycerol-based fermentation conditions (nitrogen and phosphate levels), significantly enhancing antifungal activities against *Neurospora crassa*, primarily attributed to increased production of the secondary metabolites. Thus, glycerol not only provides carbon and energy but also modulates secondary metabolism, establishing *B. subtilis* as a valuable microbial platform for antifungal compound production.

### Streptomyces clavuligerus

*Streptomyces clavuligerus* naturally synthesizes the β-lactamase inhibitor clavulanic acid (CA) using glycerol as a primary carbon source. Saudagar and Singhal (2007) showed that optimized glycerol supplementation (10–20 g/L) in fed-batch fermentations greatly enhanced CA production. Genetic evidence from Li and Townsend (2006) confirmed glycerol kinase (*glpK1*) as crucial for CA biosynthesis, reinforcing glycerol’s metabolic significance.

### Ashbya gossypii

The filamentous fungus *Ashbya gossypii* metabolizes glycerol predominantly via the G3P pathway. Traditionally employed for riboflavin production, recent metabolic engineering efforts expanded its capabilities to produce the nutraceutical intermediate orotic acid from crude glycerol, achieving titers over 4 g/L under nitrogen-limited conditions (Silva et al. 2022). This demonstrates its versatility in converting waste glycerol into valuable compounds.

### Aspergillus terreus

*Aspergillus terreus* naturally produces lovastatin, a polyketide secondary metabolite with clinical significance. Notably, the use of glycerol as a carbon source in solid-state fermentation significantly enhanced lovastatin production to 39.6 mg/L, compared to36.8 mg/L in glucose-based cultures, demonstrating glycerol’s potential to promote secondary metabolite synthesis (Subhan et al. 2016). More recently, Hasan et al. (2018) employed metabolic engineering strategies to further increase lovastatin titers under glycerol-based conditions. Overexpression of acetyl-CoA carboxylase boosted malonyl-CoA availability, enhancing polyketide precursor supply. In a subsequent study, the same group suppressed the competing (+)-geodin pathway, redirecting flux toward lovastatin biosynthesis and improving overall yield (Hasan et al. 2019). These findings illustrate the synergistic role of glycerol fermentation and genetic modification in optimizing secondary metabolite production in filamentous fungi.

In summary, among the microbial platforms that have been explored for glycerol-based secondary metabolite production, *K. phaffii* and *Y. lipolytica* show the highest titers and versatility, whereas bacterial and filamentous fungal hosts provide new directions for development. Therefore, the choice of platform must be tailored to requirements such as the desired product, process conditions, and scalability.

#### Metabolic pathways and engineering

The biosynthesis of secondary metabolites from glycerol in microbial systems depends on the precise orchestration of metabolic pathways that connect the central carbon metabolism with specialized biosynthetic routes. These pathways may be enhanced through metabolic engineering to increase the flux toward the desired compounds, improve cofactor availability, and relieve regulatory bottlenecks. This section introduces the core metabolic transformations in glycerol assimilation and their intersection with secondary metabolite biosynthesis with focus on shikimate- and MVA -derived compounds and redox-intensive lipids.

**Fig. 1:**
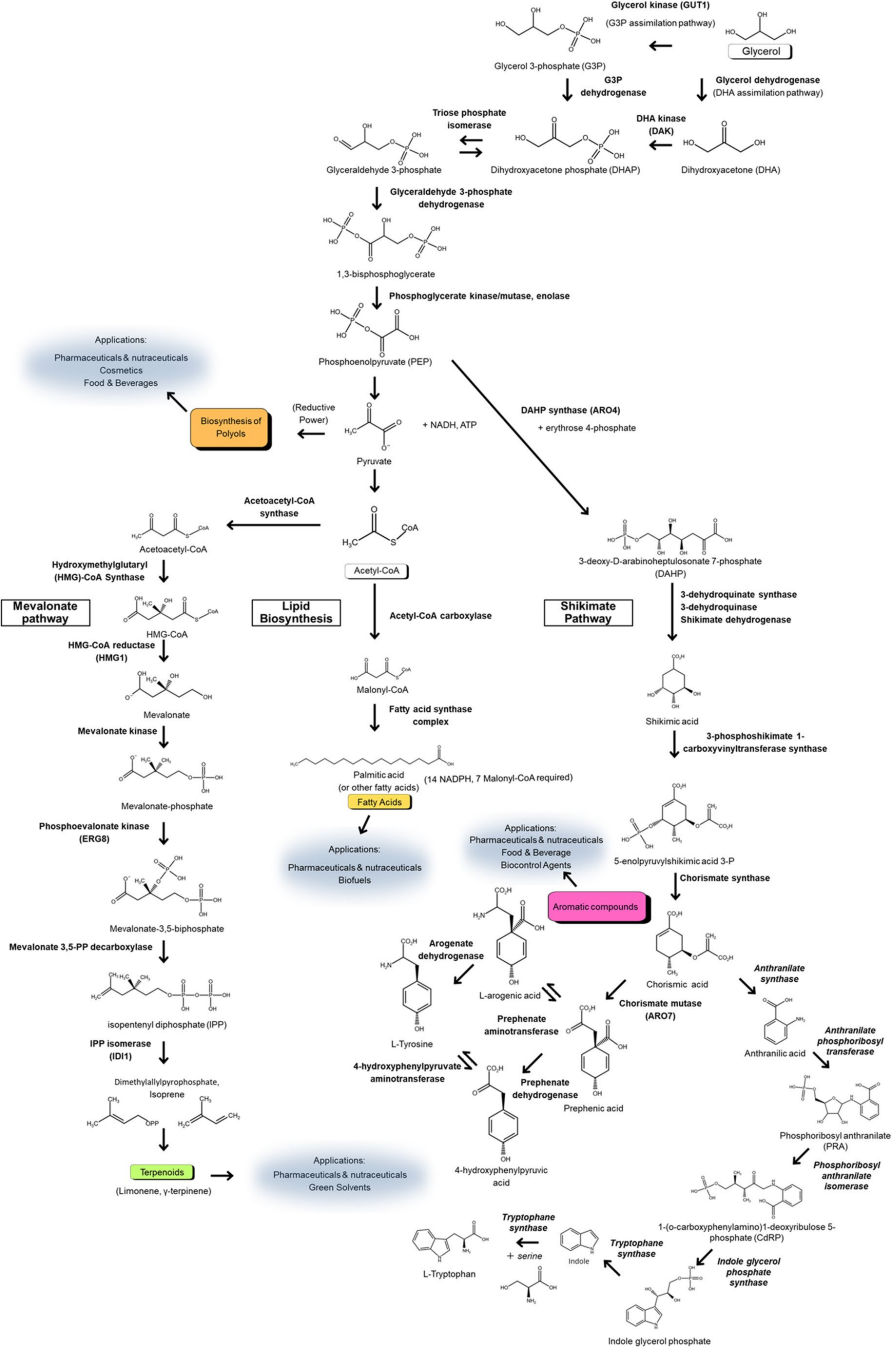
Metabolic pathways of glycerol assimilation and secondary metabolite biosynthesis. This schematic illustrates the metabolic routes for glycerol assimilation into central microbial metabolism and their channeling into secondary metabolite biosynthesis. Glycerol is converted into DHAP through either the G3P or DHA pathway, followed by integration into glycolysis. The key metabolic nodes—including the shikimate, mevalonate (MVA), and lipid biosynthesis pathways—are highlighted in boxes. In particular, the shikimate pathway is shown branching toward the biosynthesis of aromatic amino acids and their derivatives. The map also depicts the generation of key precursors such as acetyl-CoA, malonyl-CoA, and NADPH that feed into these biosynthetic pathways. Downstream applications of the resulting secondary metabolites—as pharmaceuticals, nutraceuticals, biofuels, cosmetics, green solvents, and biocontrol agents—are indicated in blue patches. The pathway topology and metabolite placement were adapted in part from publicly available resources such as the KEGG PATHWAY database (Kanehisa et al. 2021), integrated with information from cited literature

The key metabolic pathways involved in glycerol assimilation and secondary metabolite biosynthesis are summarized in Fig. 1. This schematic highlights the conversion of glycerol into glycolytic intermediates and its subsequent channeling into secondary metabolite pathways such as those of shikimate-derived aromatics, MVA-derived terpenoids, and lipids. By providing a comprehensive overview of the metabolic landscape, this figure provides a deeper understanding of the integration of glycerol metabolism with secondary metabolite production.

### Glycerol assimilation and central carbon flux

Glycerol is metabolized via the G3P and DHA pathways. In the G3P route, glycerol is phosphorylated by glycerol kinase (GUT1) to form G3P, which is oxidized by mitochondrial GUT2 to DHAP. In the DHA pathway, glycerol dehydrogenase oxidizes glycerol to DHA, which is phosphorylated by DAK to form DHAP (da Silva et al. 2009). In *K. phaffii*, glycerol is efficiently catabolized via the G3P pathway. Tomàs-Gamisans et al. (2019) performed ^13^C metabolic flux analysis and revealed that in *K. phaffii*, glycerol is primarily catabolized through glycolysis and the TCA cycle, with only minimal carbon flux through the glyoxylate shunt. This routing avoids the carbon-sparing glyoxylate pathway and instead promotes full oxidation of carbon via the TCA cycle, thereby enhancing the activity of NADPH-generating enzymes such as NADP⁺-dependent isocitrate dehydrogenase. Although the oxidative pentose phosphate pathway remains underutilized under glycerol conditions, these auxiliary TCA-based reactions provide sufficient NADPH to support anabolic activities, including secondary metabolite biosynthesis.

In filamentous fungi such as *Aspergillus terreus*, glycerol similarly serves as a favorable carbon source. Hasan et al. (2018, 2019) showed that overexpressing acetyl-CoA carboxylase increased malonyl-CoA availability and enhanced lovastatin production, while deleting the competing (+)-geodin biosynthetic pathway redirected flux toward polyketide synthesis. These findings underscore how engineering central carbon flux and precursor supply can synergize with glycerol metabolism to boost secondary metabolite yields.

### Shikimate pathway and aromatic compound production

The shikimate pathway starts with phosphoenolpyruvate (PEP) and erythrose 4-phosphate (E4P), ultimately leading to the biosynthesis of aromatic amino acids and their derivatives. In *K. phaffii*, the key initial step is catalyzed by DAHP synthase (ARO4), which condenses PEP and E4P to form 3-deoxy-D-arabinoheptulosonate 7-phosphate (DAHP). The next committed step toward phenylalanine and tyrosine synthesis is catalyzed by chorismate mutase (ARO7), which converts chorismate to prephenate. Kumokita et al. (2023) engineered *K. phaffii* to express feedback-resistant variants of these enzymes—ARO4^K229L^ and ARO7^G141S^—to boost carbon flux through the shikimate pathway, enhancing the biosynthesis of resveratrol, naringenin, and *p*-coumarate. Supplementing cultures with aspartate and tryptophan further increased the levels of intermediates such as 3-dehydroquinate, 3-dehydroshikimate, and shikimate, indicating a metabolic synergy between carbon and nitrogen pathways. Compared to glucose, glycerol provides a more reduced carbon source, enhancing intracellular NADPH generation. Since the shikimate pathway is NADPH-intensive, this redox advantage makes glycerol particularly effective. Under glycerol-based cultivation with amino acid supplementation, titers of *p*-coumarate, naringenin, and resveratrol reached 92, 297, and 452 mg/L, respectively—representing 2.0-, 2.2-, and 2.6-fold increases compared to glucose-based cultures (Kumokita et al. 2022, 2023). This indicates the synergistic effect of glycerol metabolism and amino acid supplementation on the production of shikimate pathway–derived compounds in *K. phaffii*.

### MVA pathway and terpenoid biosynthesis

Terpenoids such as limonene are synthesized via the MVA pathway. *Y. lipolytica* has been engineered to overproduce monoterpenes from glycerol. Cao et al. (2016) overexpressed certain key MVA pathway genes, including *HMG1* (encoding HMG-CoA reductase), *IDI1* (encoding isopentenyl-diphosphate isomerase), and *ERG8* (encoding phosphomevalonate kinase), and a codon-optimized limonene synthase gene from *Mentha* sp. The final engineered strain produced 23.6 mg/L limonene in shake flask cultures, representing a 226-fold increase compared with the baseline. This yield was further improved by applying a dodecane overlay, forming a two-phase cultivation system in which the hydrophobic dodecane phase captured the volatile product, reduced feedback inhibition, and facilitated product recovery. This study highlights the importance of balancing pathway expression with effective product removal in terpenoid engineering.

Terpenoid biosynthesis requires abundant NADPH and acetyl-CoA. Glycerol metabolism enhances both, offering a superior carbon and redox supply. *S. cerevisiae* strains genetically engineered to produce plant-derived ginsenosides from glycerol-based media yielded up to 384.52 mg/L compound K (a ginsenoside derivative), demonstrating the suitability of glycerol for heterologous terpenoid production (Nan et al. 2020).

### Lipid, polyol, and organic acid biosynthesis

Oleaginous yeasts such as *Y. lipolytica* redirect carbon flux from biomass formation to the synthesis of lipids and polyols under nitrogen-limited conditions. The high reduction degree of glycerol makes it an ideal carbon source for NADPH-intensive biosynthetic processes such as fatty acid production. Papanikolaou et al. (2017a) reported lipid accumulation exceeding 50% of DCW in *Y. lipolytica* cultivated on crude glycerol. Additionally, polyols such as erythritol and mannitol were produced at titers over 80 g/L and 50 g/L, respectively, by wild-type strains of *Y. lipolytica* under osmotic stress. (Rywińska et al. 2013; Ziuzia et al. 2023). Citric acid, a primary metabolite, has also been produced at over 100 g/L in some strains. While these metabolites are primarily associated with primary metabolism, their biosynthetic pathways rely on redox cofactor availability and stress-responsive regulation, offering parallels to secondary metabolite production. These examples indicate how glycerol supports reductive anabolic processes, providing insights that may inform strategies for engineering more complex secondary metabolic pathways.

### Regulation of secondary metabolism via redox homeostasis

In addition to enzyme engineering, secondary metabolism is controlled by regulatory mechanisms. Santamaria et al. (2022) studied *Pseudomonas aeruginosa* and found that rhamnolipid biosynthesis depends less on the presence of genes than on the ability of the cell to manage oxidative stress. Under glycerol-fed conditions, only the strains with sufficient antioxidant capacity produced rhamnolipids. This phenomenon known as “metabolic prudence” suggests that microbes prioritize primary metabolism unless sufficient resources and redox balance support secondary metabolite synthesis. Thus, future engineering efforts must go beyond enzyme expression and incorporate strategies for maintaining NADPH/NADP⁺ balance, glutathione metabolism, and oxidative stress responses to unlock biosynthetic potential from glycerol.

## Bioprocess design and optimization

The success of glycerol-based secondary metabolite production depends as much on the choice of microbial host and genetic modifications as on the optimization of fermentation conditions such as medium composition, aeration, feeding strategy, and pH control. Effective bioprocess design enables the redirection of carbon flux toward product synthesis while minimizing byproducts and improving yield, titer, and productivity.

### Culture conditions and nutrient balance

The carbon to nitrogen (C/N) ratio is a crucial factor in secondary metabolite production as it influences metabolic shifts from growth to biosynthesis. For example, *Y. lipolytica* favored lipid and polyol accumulation from glycerol under nitrogen limitation (Papanikolaou et al. 2017a). Similarly, *B. subtilis* produced secondary antifungal metabolites such as lipopeptides from glycerol in the presence of optimized nitrogen and phosphate levels. Trivunović et al. (2022) have shown that the highest antifungal activity against *Neurospora crassa* was achieved using a medium containing 49.68 g/L glycerol, 2.90 g/L sodium nitrite, and 6.49 g/L dipotassium phosphate, which highlights the importance of nutrient balancing in triggering secondary metabolism.

Kumokita et al. (2023) showed that supplementation of glycerol-based media with amino acids such as aspartate and tryptophan enhanced shikimate pathway activity in *K. phaffii*, leading to improved production of aromatic compounds including 92 mg/L *p*-coumarate, 297 mg/L naringenin, and 452 mg/L resveratrol after 96 h of flask cultivation. This enhancement is likely due to three factors. First, aspartate serves as a precursor for multiple biosynthetic pathways (e.g., lysine, threonine, and nucleotide synthesis), reducing biosynthetic burden and allowing greater carbon flux through the shikimate pathway. Second, tryptophan supplementation may alleviate feedback inhibition of key enzymes such as DAHP synthase or chorismate mutase, thereby increasing metabolic flux upstream. Additionally, supplying end products exogenously helps stabilize internal metabolite pools and facilitates efficient routing of carbon to desired secondary metabolites (Kumokita et al. 2022, 2023).

### Aeration and oxygen transfer

Oxygen availability exerts a substantial effect on glycerol metabolism. Generally, aerobic conditions are required for efficient glycerol utilization via oxidative phosphorylation and biosynthesis of several reduced secondary metabolites. For example, the agitation rate and dissolved oxygen level determined the balance between polyol production (e.g., erythritol) and lipid accumulation (Papanikolaou et al. 2017a) in *Y. lipolytica*. Cao et al. (2016) showed that oxygen supply and the use of a dodecane overlay significantly enhanced limonene production in *Y. lipolytica* cultivated in shake flasks. The two-phase cultivation system utilized dodecane as an in situ organic extractant to capture the volatile limonene and reduce feedback inhibition. This highlights the importance of phase separation strategies for recovering hydrophobic or toxic secondary metabolites during glycerol-based fermentation (Cao et al. 2016).

### Glycerol feeding strategies

Continuous and fed-batch fermentation modes allow the dynamic control of glycerol concentration, prevent substrate inhibition, and enable staged metabolic transitions. Nan et al. (2020) applied a glycerol–ethanol co-feeding strategy to improve UDP-glucose availability and glycosylation efficiency in engineered *S. cerevisiae*, which led to a 45.7% increase in compound K production—a deglycosylated ginsenoside with pharmacological activity derived from *Panax* plants—and a final titer of 1.7 g/L under bioreactor conditions. In *K. phaffii*, a conventional strategy for recombinant protein production involves a two-phase cultivation process—initial biomass accumulation using glycerol, followed by induction with methanol to activate the AOX1 promoter (Cregg et al. 2000; Celik and Calık 2012). However, for the production of metabolites that do not require AOX1-dependent gene expression, glycerol can be maintained as the carbon source throughout the culture period. This approach enables a more stable and controllable carbon flux into secondary metabolite pathways, as demonstrated in recent studies on aromatic compound production from glycerol (Kumokita et al. 2023). Moreover, Zacharopoulos and Theodoropoulos (2023) proposed that continuous feeding strategies, combined with cell-recycling bioreactor systems and computational modeling, can improve the overall carbon conversion efficiency and maintain steady-state metabolism. Although their work focused on succinic acid, their methodological framework for maintaining redox and flux stability could also inform process optimization for glycerol-based secondary metabolite production under controlled bioprocessing conditions.

### Process monitoring and control

Advances in bioprocess analytics such as the online monitoring of pH, dissolved oxygen tension, and carbon source concentrations are becoming increasingly important for the dynamic control of secondary metabolism. The integration of real-time omics data and biosensors may lead to promising responsive control strategies that optimize metabolite production during key physiological transitions such as the shift from exponential growth to stationary-phase biosynthesis. Additionally, recent developments in label-free monitoring technologies have enabled the noninvasive assessment of cellular physiological states. For example, morphological metrics such as cell size and volume measured using Coulter counters may be rapid indicators of the metabolic shift from proliferation to product synthesis (Kamasaka et al. 2025, submitted). This approach offers a scalable and automation-compatible tool for next-generation process control.

## Crude glycerol valorization

### Composition and challenges

Crude glycerol is a major byproduct of biodiesel production that is an abundant and low-cost carbon source for microbial fermentation. However, its application presents several challenges owing to the presence of impurities such as methanol, free fatty acids, salts, soap residues, water, and unreacted triglycerides (da Silva et al. 2009; Samul et al. 2014). The concentrations and compositions of these contaminants vary depending on the feedstock, catalyst, and transesterification processes used (Kumar et al. 2019). These impurities may inhibit microbial growth, disrupt membrane integrity, and impair enzymatic activity, which ultimately reduce fermentation efficiency. Particularly, methanol and salt contents are often identified as major inhibitory factors. Moreover, the high variability in crude glycerol composition requires strategies for reliable and reproducible fermentation performance such as thorough feedstock characterization, optimized feeding strategies, and minimal pretreatment in some cases to mitigate toxicity. Nevertheless, the successful microbial valorization of crude glycerol is a promising route for advancing circular bioeconomic approaches by converting industrial waste into high-value secondary metabolites.

### Microbial tolerance and bioconversion

Despite these challenges, several microorganisms have shown the ability to effectively tolerate and metabolize crude glycerol. For example, *Y. lipolytica* has been widely studied for its ability to utilize crude glycerol as a substrate to produce lipids, polyols, and organic acids. Papanikolaou et al. (2017a) and Sayin Börekçi et al. (2022) have reported high yields of erythritol and mannitol under nitrogen-limited conditions with lipid accumulation exceeding 50% of the cell dry weight. Rywińska et al. (2024) have engineered *Y. lipolytica* strains to co-produce citric acid and erythritol. These showed titers of > 140 g/L even when unrefined glycerol was used as the substrate. Additionally, Ziuzia et al. (2023) have isolated wild-type strains of *Y. lipolytica*, *Candida magnoliae*, and *Starmerella magnoliae* that are capable of producing citric acid, mannitol, and erythritol directly from crude glycerol without detoxification. Additionally, bacterial hosts have shown promise in glycol utilization. *B. subtilis* has been used to produce secondary antifungal metabolites from crude glycerol after the careful optimization of the medium. Trivunović et al. (2022) have shown that adjusting the phosphate and nitrogen levels enabled high bioactivity against *N. crassa* despite the presence of impurities. These findings highlight the importance of both strain robustness and medium engineering for crude glycerol valorization.

### Purification and pretreatment strategies

Often pretreatment or purification of crude glycerol is necessary to reduce the levels of impurities such as methanol, salts, soaps, and matter organic non-glycerol (MONG), which inhibit microbial growth and enzyme activity (Attarbachi et al. 2023). Common methods to achieve this aim include acidification and filtration to remove soaps, solvent extraction to remove hydrophobic impurities, and vacuum distillation to remove methanol. Adsorption- and membrane-based techniques are additional options for polishing and desalination. These methods vary in complexity and cost and must be selected based on the sensitivity of the microbial host and desired product. The use of robust strains minimizes the number of required pretreatments (e.g., methanol removal only). However, the targeting of high-value metabolites and operating under tightly controlled industrial conditions justify extensive purification.

## Industrial applications

Secondary metabolites derived from glycerol-fed microbial systems exhibit promising potential across multiple industrial sectors, including pharmaceuticals, food and beverage, biofuels, cosmetics, and agriculture. Utilizing glycerol as a feedstock not only supports the circular economy by valorizing biodiesel byproducts but also aligns with green chemistry principles due to its renewable and reduced nature.

### Pharmaceuticals and nutraceuticals

Aromatic compounds such as resveratrol, naringenin, and *p*-coumarate produced through engineered microbial systems—exhibit a range of bioactivities including anti-inflammatory, antioxidant, and anticancer effects (Kumokita et al. 2023). These molecules are being explored for use in functional foods and therapeutic supplements. In parallel, ginsenoside derivatives (e.g., compound K), produced by engineered yeasts, are gaining attention in the pharmaceutical industry for their adaptogenic and immunomodulatory properties, particularly in the North American and Asian markets (Nan et al. 2020).

### Food and beverage industry

Polyols such as erythritol and mannitol, commonly used as low-calorie sweeteners, can be microbially produced from crude glycerol using *Y. lipolytica* and red yeasts (Papanikolaou et al. 2017a; Ziuzia et al. 2023). This approach provides an attractive alternative to sugar-based fermentations, with economic and sustainability advantages. Likewise, organic acids such as citric acid are widely used in food preservation and flavoring and can be produced efficiently from glycerol by oleaginous yeasts. Although filamentous fungi like *Aspergillus niger* remain the primary industrial producers, yeasts such as *Y. lipolytica* have also indicated considerable potential for citric acid production under nitrogen-limited conditions (Li and Townsend 2006).

### Biofuels and green solvents

Oleaginous yeasts such as *Y. lipolytica* and *R. toruloides* metabolize glycerol to accumulate > 50% of their dry cell weight in lipids during the production of single-cell oils, which may be directly converted to biodiesel or oleochemicals (Beopoulos et al. 2008). These novel routes of oil production may be advantageous for glycerol-to-lipid biorefineries, especially for integration with existing biodiesel infrastructure. Monoterpenes such as limonene that are synthesized from glycerol by engineered *Y. lipolytica* strains have shown potential as green solvents, biofuels, and precursors of biodegradable polymers (Cao et al. 2016). Although the titers remain modest, the use of non-toxic feedstocks and robust hosts are conducive for the scale-up of these systems.

In summary, the wide range of applications of glycerol-derived secondary metabolites reinforces their strategic importance in building resilient low-carbon manufacturing systems. Furthermore, the continued advancement of fermentation technology and strain engineering would expand the scope of viable products and host platforms. Thus, glycerol plays a significant role as a cornerstone feedstock in industrial biotechnology.

## Challenges and future directions

Despite significant progress in harnessing glycerol for microbial secondary metabolite production, several scientific and technical challenges remain. Addressing these limitations is crucial for harnessing the full potential of glycerol-fed bioprocesses for a range of industrial applications.

### Strain-specific metabolic constraints

Not all industrially relevant hosts are capable of metabolizing glycerol to the same extent. Although non-conventional yeasts such as *K. phaffii* and *Y. lipolytica* show high glycerol assimilation efficiency, organisms such as *S. cerevisiae* and *E. coli* often exhibit slow growth, poor product yields, or require substantial genetic rewiring for glycerol utilization (Xiberras et al. 2019). Furthermore, an incomplete knowledge of carbon flux regulation and cofactor dynamics can constrain productivity even in strains engineered for glycerol metabolism. Thus, comprehensive fluxomic, proteomic, and metabolomic analyses are required to identify hidden bottlenecks and regulatory mechanisms in central and secondary metabolism.

### Variability and toxicity of crude glycerol

The composition of crude glycerol varies depending on the feedstock, biodiesel production conditions, and impurities such as methanol, soaps, salts, and free fatty acids. These components negatively affect cell viability, enzyme activity, and fermentation kinetics. Although some robust strains tolerate these conditions, several require adaptation or process modifications. Therefore, the development of standardized pretreatment protocols, engineering of tolerance traits, or usage of microbial consortia to partition the detoxification and production tasks may be required to overcome this variability.

### Nutrient regulation and co-feeding strategies

Secondary metabolism is strictly regulated by nitrogen, phosphate, and cofactor availability. Optimal carbon-to-nitrogen (C/N) ratio, amino acid supplementation, and dynamic micronutrient feeding are often necessary to shift the metabolic flux from growth to product formation. However, few generalizable rules exist across hosts and product types. Recent findings such as the effect of tryptophan and aspartate on shikimate pathway activation (Kumokita et al. 2023) emphasize the need for host- and product-specific nutrient optimization. Future studies must prioritize sensor-based feedback-controlled feeding strategies to dynamically guide these transitions.

### Genetic tools and pathway engineering

Although CRISPR/Cas9 and modular pathway assembly have accelerated metabolic engineering, challenges remain with regard to balancing gene expression, managing toxic intermediates, and maintaining plasmid stability—particularly in non-model organisms. Advanced methods such as synthetic regulatory circuits, cofactor channeling, and chassis optimization (e.g., minimizing competing pathways or protease activity) are required to support high-yield production in glycerol-fed systems. Additionally, increasing the number of genome-scale models tailored to glycerol metabolism—such as the PpaCore model developed for *K. Phaffii* (Tomàs-Gamisans et al. 2019)—may improve design accuracy.

### Process integration and scale-up

Laboratory-scale results do not always translate into industrial success. Issues such as oxygen transfer, foam formation, product volatility, and downstream separation need to be addressed for scaling up. In fact, strategies such as two-phase extraction or in situ product removal are necessary in the case of volatile or hydrophobic products such as limonene (Cao et al. 2016).

### Expanding product scope

Most current studies have focused on polyols, aromatics, lipids, and organic acids. However, the reducing power and metabolic flexibility of glycerol make it a promising feedstock for the production of alkaloids, terpenoids, polyketides, and glycosylated compounds, which remain underexplored in microbial systems. Future studies are needed to investigate pathway modularity, cocultivation platforms, and chassis tailoring to expand the scope of glycerol-accessible products.

In summary, although glycerol offers numerous advantages as a feedstock for secondary metabolite production, further advances in strain development, bioprocess engineering, and systems-level metabolic understanding are essential for harnessing its full industrial potential. Additionally, the integration of synthetic biology, real-time process monitoring, and metabolic modeling to design robust high-performance microbial platforms tailored for glycerol-based bioproduction are required for the optimal usage of glycerol for industrial applications.

## Conclusion

Glycerol has emerged as a versatile and sustainable carbon source for the microbial production of secondary metabolites, offering advantages such as high reduction degree, low cost, and broad industrial availability. This review has highlighted diverse microbial platforms—including yeasts (*K. phaffii*, *Y. lipolytica*, *S. cerevisiae*), filamentous fungi (*A. gossypii*, *A. terreus*), red yeasts (*R. toruloides*), and bacteria (*B. subtilis*, *S. clavuligerus*)—that utilize glycerol either natively or in engineered forms to synthesize a wide range of high-value products.

Secondary metabolites such as resveratrol, naringenin, *p*-coumarate, mevinolinic acid, clavulanic acid, and terpenoids have been successfully produced from glycerol, often through targeted metabolic engineering. In contrast, certain wild-type strains efficiently produce polyols, lipids, and antibiotics under stress-inducing or nutrient-limited conditions. These case studies collectively illustrate how glycerol metabolism intersects with secondary metabolite biosynthesis via pathways like the shikimate and MVA routes, both of which benefit from enhanced NADPH availability and precursor supply.

Importantly, while many examples rely on genetically modified strains, non-engineered hosts also show considerable potential for secondary metabolite production from crude or pure glycerol. The choice of host and strategy must therefore be tailored to the desired compound, scalability, and regulatory context. Future research should focus on optimizing redox balance, improving bioreactor operation strategies, and expanding the range of microbial hosts to fully exploit glycerol’s potential in biomanufacturing. Taken together, glycerol represents a promising feedstock for the next generation of sustainable, microbially produced secondary metabolites.

## Data Availability

No datasets were generated or analysed during the current study.
